# Analysis of risk factors for non-alcoholic fatty liver disease in hospitalized children with obesity before the late puberty stage

**DOI:** 10.3389/fendo.2023.1224816

**Published:** 2023-08-31

**Authors:** Lishan Zhou, Linli Zhang, Lingling Zhang, Wei Yi, Xue Yu, Hong Mei, Haiyan Xiao, Yuji Wang, Huan Qin, Xiaoli Xiong, Suqi Yan, Hui Dong, Peng Chen, Xiaohong Chen

**Affiliations:** ^1^ Wuhan Children’s Hospital (Wuhan Maternal and Child Healthcare Hospital), Tongji Medical College, Huazhong University of Science and Technology, Wuhan, Hubei, China; ^2^ Wuhan Wuchang Hospital, Wuchang Hospital Affiliated to Wuhan University of Science and Technology, Wuhan University of Science and Technology, Wuhan, Hubei, China; ^3^ Department of Pediatrics, Wuhan Children’s Hospital (Wuhan Maternal and Child Healthcare Hospital), Affiliated Hospital of Jianghan University, Jianghan University, Wuhan, Hubei, China; ^4^ Institute of Integrated Traditional Chinese and Western Medicine, Tongji Hospital, Tongji Medical College, Huazhong University of Science and Technology, Wuhan, Hubei, China

**Keywords:** nonalcoholic fatty liver disease, children, risk factors, obese, prepuberty, early puberty

## Abstract

**Objective:**

This study aimed to determine the clinical characteristics of obese pediatric non-alcoholic fatty liver disease (NAFLD) in central China and verify the applicability of some known risk factors for pediatric NAFLD before late puberty.

**Methods:**

This was a retrospective case–control study. A total of 1,029 inpatients at Wuhan Children’s Hospital before the late puberty stage were enrolled in the study, including 815 children with obesity (non-NAFLD group) and 214 children with obesity and NAFLD (NAFLD group) diagnosed by liver ultrasound. Subgroup analyses were performed according to sex and puberty. The anthropometric indices and laboratory test data of these 1,029 children were sorted. After intergroup comparison, a logistic regression model was used to determine the risk factors for pediatric NAFLD. Significant risk factors for NAFLD were further tested using receiver operating characteristic (ROC) curves to evaluate their ability to predict an early diagnosis of NAFLD.

**Results:**

The NAFLD group had a mean age of 11.03 ± 1.66, with 11.18 ± 1.66 and 10.27 ± 1.45 years for male and female children, respectively (*p* < 0.05 and *p* < 0.01, respectively). Even subdivided by both sex and puberty, raised body mass index (BMI), homeostatic model-insulin resistance, triglycerides, alanine transaminase (ALT), aspartate aminotransferase (AST), and gamma-glutamyl transferase (γ-GT) were still found in the non-NAFLD and NAFLD groups (*p* < 0.05 and *p* < 0.01, respectively). The results of logistic regression analysis showed that BMI (odds ratio [OR], 1.468;95% confidence interval [CI], 1.356-1.590; *p*<0.001) and ALT (OR, 1.073;95%CI, 1.060-1.087; *P*<0.001) were two most independent risk factors for NAFLD. The maximal OR for BMI was 1.721 (95% CI, 1.336–2.217). In the female group, the maximal OR of ALT was found to be 1.104 (95% CI, 1.061–1.148). Age and thyroid-stimulating hormone (TSH) and γ-GT levels were also risk factors, but they appeared only in some groups. The results of the ROC analysis showed that ALT was a better predictor of pediatric NAFLD than BMI. The maximum area under the ROC curve in six of the nine groups belongs to ALT.

**Conclusions:**

BMI, ALT, and age are risk factors for NAFLD in children with obesity before late puberty. BMI had the greatest exposure risk for NAFLD, and ALT had the highest predictive value for the diagnosis of NAFLD. At the stratified level, for exposure risk, age was specific to the male sex, TSH was specific to the early puberty stage, and γ-GT was specific to the female sex plus the prepuberty stage. On a stratified level, for the female sex, even with age stratification, BMI rather than ALT has a better ability for the diagnosis of NAFLD.

## 1 Introduction

Not only in developed countries but also in China, non-alcoholic fatty liver disease (NAFLD) is the most common cause of chronic liver disease in children and adolescents (1–5). Recent data report a prevalence of NAFLD of 3%–10% in children worldwide and 3.4% in China (1–5). Pediatric NAFLD is defined as chronic liver steatosis (fat content in the hepatocytes >5%) in children and adolescents aged <18 years, which is a metabolic stress liver injury closely related to insulin resistance and genetic susceptibility. However, the clinical pathological syndrome of chronic fatty deposition in the liver caused by alcohol and other definite pathogenic factors should be excluded (3). The spectrum of pediatric NAFLD includes non-alcoholic fatty liver (NAFL), non-alcoholic steatohepatitis, related liver fibrosis, and cirrhosis (3).

Most pediatric patients with NAFLD are in the NAFL stage and are easily ignored owing to the lack of clinical symptoms. The development and progression of the disease in childhood lead to an increased risk of long-term morbidity, especially cardiovascular diseases (6). Therefore, reliable forecasting factors are necessary for early screening. Obesity is a leading risk factor for pediatric NAFLD. The prevalence of NAFLD in children with obesity and those who are overweight has increased to 50%–80% (7, 8). Male sex is also a risk factor for NAFLD in both children and adults (9, 10). Additionally, the prevalence of NAFLD in children and adolescents increases with age, showing a growth rate of 0.7% in children 2 to 4 years old to 7% in teenagers (6).

However, epidemiological data from China are mostly from the Yangtze River Delta region. Therefore, the first aim of this study was to determine the clinical characteristics of pediatric obese patients with NAFLD in Central China. Owing to the influence of age on the prevalence rate, there were relatively few cases in the pre- and early puberty stages. Thus, the secondary aim of this study was to verify the applicability of known risk factors for pediatric NAFLD before late puberty.

## 2 Materials and methods

### 2.1 Study population

This cross-sectional study collected inpatient clinical data from the Department of Endocrinology and Metabolism at Wuhan Children’s Hospital between January 2014 and December 2021. Based on the database of patient files in the Wuhan Children’s Hospital, 305 patients aged between 7.17 and 16.58 years were diagnosed with both obesity and NAFLD and were discharged during the observation period. Simultaneously, during the aforementioned period, through random sampling, 1,233 patients aged between 3.00 and 17.17 years were discharged and diagnosed with obesity but without NAFLD. After excluding patients in the late puberty stage and those with incomplete data, the clinical data of 214 children with obesity and NAFLD (the NAFLD group) were collected, and those of 815 children with obesity and without NAFLD (the non-NAFLD group) were randomly selected and analyzed retrospectively. Meanwhile, subgroup analysis was performed according to sex and puberty: <10.65 (P_50_) years as male prepuberty, between 10.65 (P_50_) and 14.83 (P_50_) years as male early puberty, <9.65 (P_50_) years as female prepuberty, and between 9.65 (P_50_) and 12.91 (P_50_) years as female early puberty (11).

The inclusion criteria for pediatric obesity were based on the body mass index (BMI) classification criteria for overweight and obesity screening of Chinese children and adolescents according to age and sex (12). The inclusion criteria for pediatric NAFLD were based on an imaging diagnosis using ultrasonography (2, 13).

The exclusion criteria were patients with secondary obesity (such as hypothyroidism, Cushing’s disease, or hypothalamic obesity) and patients with other specific causes of fatty liver: a) genetic-metabolic causes: Turner syndrome, α- and β-oxidation defects, low β lipoproteinemia, Wilson’s disease, alpha-1-antitrypsin (AAT) deficiency, abetalipoproteinemia or hypobetalipoproteinemia, citrin deficiency, fatty acid metabolism disorder, congenital disorders of glycosylation, cystic fibrosis, Shwachman-Diamond syndrome, familial hyperlipoproteinemias, glycogen storage disease (types  I, VI, and IX), fructosemia, porphyria cutanea tarda, mitochondrial and peroxisomal defects, organic acidosis, and homocystinuria; b) general or systemic causes: thyroid disorders, celiac disease, diabetes mellitus type 1, hepatitis C, hypothalamic–pituitary disorders, inflammatory bowel disease, autoimmune liver disease, protein-calorie malnutrition, rapid weight loss, and anorexia nervosa; c) drugs or chemicals causes: estrogens, corticosteroids, diltiazem, nifedipine, cocaine, ethanol, methotrexate, valproate, pesticides, vitamin A, zidovudine, and human immunodeficiency virus treatments; secondary obesity caused by heredity or endocrine diseases, etc.

This study was approved by the Medical Ethics Committee of the Wuhan Children’s Hospital (Approval No. 2022R051-E01).

### 2.2 Measurements

Pediatricians in the endocrinology department performed all the anthropometric and blood pressure measurements.

The height was measured to the nearest 0.1 cm. Similarly, body weight was measured to the nearest 0.1 kg. BMI was calculated as body weight (kg) divided by height (m) squared.

A mercury sphygmomanometer was used to measure systolic blood pressure (SBP; mmHg) and diastolic blood pressure (DBP; mmHg) in the right upper arm after resting for at least 10 min. Blood pressure was assessed three times at 2-minute intervals, and the mean of these three measurements was calculated. The blood pressure classification of children was based on their sex, age, and height (14, 15): normal, SBP and DBP <P_90_; hypertension, SBP or DBP between P_95_ and P_99_; and severe hypertension, SBP or DBP >P_99_.

### 2.3 Laboratory tests

Blood samples were collected after fasting overnight for 10 hours. All blood samples were processed, refrigerated, and transported to the clinical laboratory of Wuhan Children’s Hospital and analyzed as soon as possible. All clinical analyses were performed in our clinical laboratory, which is certified by the China National Accreditation Service for Conformity Assessment.

The serum concentrations of general biochemistry tests were measured using the Cobas c 702 analyzer (Roche, Mannheim, Germany), such as triglyceride (TG), total cholesterol (TC), high-density lipoprotein cholesterol (HDL-C), low-density lipoprotein cholesterol (LDL-C), fasting plasma glucose, and liver chemistry, for instance, alanine transaminase (ALT), aspartate aminotransferase (AST), gamma-glutamyl transferase (γ-GT), alkaline phosphatase (ALP), albumin (ALB), and globulin (GLB). Routine blood tests were performed using the XN-3000 instrument (SYSMEX, Kobe, Japan). These parameters included white blood cells (WBC), red blood cells (RBC), hemoglobin (Hb), platelet count, neutrophils (NEU), lymphocytes (LYM), and monocytes (MONO). Fasting insulin was examined using an IMMULITE 2000 XPi Immunoassay (Siemens, Munich, Germany), and glycated hemoglobin (HbA1c) was measured using a D-10 glycosylated hemoglobin meter (Bio-Rad, Hercules, CA, USA). Thyroid function tests, including serum triiodothyronine (T3), thyroxine (T4), thyroid-stimulating hormone (TSH), free T3 (FT3), and free T4 (FT4) levels, were performed using the ADVIA Centaur XP (Siemens, Germany).

Insulin resistance was assessed using a homeostatic model (homeostasis model assessment insulin resistance index (HOMA-IR)). The formula for HOMA-IR was fasting insulin multiplied by fasting plasma glucose divided by 22.5.

### 2.4 Sonographic examination

Sonographic examinations were performed using ultrasound (EPIQ5, Philips Healthcare, Bothell, WA, USA) by two trained pediatric sonographers. The patients were instructed to fast for at least 8 hours before the examination. We performed a comprehensive and systematic scan of the liver in the supine and left lateral decubitus positions. The characteristics of enhanced echo in the front field of the liver (“bright liver”), attenuation of echo in the far field, and unclear display of intrahepatic duct structure were classified as NAFLD (3, 13, 16).

### 2.5 Other investigation contents

The birth weight (kg) of each infant was recorded. The delivery mode of the patient’s mother (natural or cesarean section) was also recorded.

### 2.6 Statistical analysis

IBM SPSS Statistics for Windows, version 21.0 (IBM Corp., Armonk, NY, USA), was used to perform the statistical analyses. Continuous variables in accordance with the normal distribution were represented as the mean ± standard deviation. Continuous variables that were not normally distributed were presented as medians (25% quartiles, 75% quartiles). Categorical variables were presented as numbers. A two-tailed t-test was used to compare continuous variables with a normal distribution across groups. The rank sum test was used to compare continuous variables that were not normally distributed across groups. The chi-square (χ^2^) test was used to compare categorical variables across groups. Risk factor analysis was performed using binary logistic regression analysis. A multiple logistic regression analysis was conducted to determine the significant predictors after controlling for all variables. Candidate risk factors for NAFLD were further tested by discrimination analysis using receiver operating characteristics (ROC). A *p*-value of <0.05 was considered statistically significant.

## 3 Results

### 3.1 Patient characteristics

Data from a total of 1,029 inpatients, including children and adolescents with obesity before the late puberty stage (581 male and 448 female patients; mean age, 9.99 ± 1.80 years), were collected. Based on liver ultrasound findings, 1,029 patients were divided into two groups: 815 patients in the non-NAFLD group with 402 male and 413 female patients (mean age, 9.50 years [range, 8.50–10.92]) and 214 in the NAFLD group with 179 male and 35 female patients (mean age, 11.17 years [range, 9.90–12.17]) (**Table 1**).

**Table 1:** Clinical characteristics in hospitalized children with obesity before the late puberty stage with or without NAFLD.

		Total		
		Non-NAFLD (n = 815)	NAFLD (n = 214)	*p*-Value
Age (year)		9.50 (8.50, 10.92)	11.17 (9.90, 12.17)	0.000
Sex (n, %)	Male	402 (49.33%)	179 (83.64%)	0.000
	Female	413 (50.67%)	35 (16.36%)	
BMI (kg/m^2^)		22.20 (20.67, 24.24)	27.60 (25.68, 29.89)	0.000
Blood pressure (n, %)	Normal	625 (76.69%)	122 (57.01%)	0.000
	Hypertension	151 (18.53%)	57 (26.64%)	
	Severe hypertension	39 (4.78%)	35 (16.35%)	
Birth weight (kg)		3.33 (3.00, 3.65)	3.40 (3.05, 3.65)	0.338
Delivery mode (n, %)	Natural	268 (32.88%)	86 (40.19%)	0.055
	Cesarean section	547 (67.12%)	128 (59.81%)	
Glucose and lipid metabolism				
HbA1c (%)		5.30 (5.10, 5.50)	5.40 (5.20, 5.60)	0.000
HOMA-IR		2.15 (1.28, 3.61)	3.81 (2.43, 5.29)	0.000
TG (mmol/L)		0.90 (0.68, 1.25)	1.24 (0.92, 1.65)	0.000
TC (mmol/L)		3.71 ± 0.66	4.02 ± 0.79	0.000
HDL-C (mmol/L)		1.23 (1.05, 1.42)	1.09 (0.93, 1.28)	0.000
LDL-C (mmol/L)		2.24 (1.84, 2.64)	2.54 (2.07, 2.95)	0.000
Blood routine				
WBC (×10^9^/L)		6.15 (5.32, 7.22)	6.68 (5.77, 7.74)	0.000
RBC (×10^12^/L)		4.59 (4.39, 4.80)	4.68 (4.48, 4.91)	0.000
Hb (g/L)		128.00 (122.00, 133.00)	130.50 (124.00, 135.00)	0.001
PLT (×10^9^/L)		286.00 (255.00, 324.00)	293.00 (256.50, 339.00)	0.110
NEU (×10^9^/L)		3.26 (2.63, 3.99)	3.51 (2.89, 4.27)	0.000
LYM (×10^9^/L)		2.33 (1.94, 2.67)	2.39 (2.01, 2.87)	0.019
MONO (×10^9^/L)		0.38 (0.32, 0.48)	0.45 (0.37, 0.53)	0.000
Liver function				
ALT (U/L)		13.00 (10.00, 17.00)	51.50 (25.00, 85.25)	0.000
AST (U/L)		20.00 (17.00, 23.00)	33.00 (22.00, 48.00)	0.000
γ-GT (U/L)		13.00 (11.00, 16.00)	28.00 (19.00, 44.00)	0.000
ALP (U/L)		252.00 (210.00, 307.00)	257.00 (216.00, 314.00)	0.413
ALB (g/L)		45.40 (43.80, 47.20)	46.00 (44.10, 48.20)	0.005
GLB (g/L)		24.51 ± 3.29	25.51 ± 3.55	0.000
Thyroid function				
T3 (nmol/L)		2.23 (1.96, 2.51)	2.21 (1.94, 2.49)	0.556
T4 (nmol/L)		105.50 ± 26.49	106.22 ± 23.86	0.722
TSH (μIU/ml)		2.34 (1.63, 3.33)	2.75 (1.85, 3.84)	0.001
FT3 (pmol/L)		6.45 (6.04, 6.95)	6.32 (5.78, 6.83)	0.002
FT4 (pmol/L)		15.89 (14.44, 17.34)	16.06 (14.40, 17.75)	0.389

NAFLD, non-alcoholic fatty liver disease; BMI, body mass index; HbA1c, glycosylated hemoglobin; HOMA-IR, homeostasis model assessment insulin resistance index; TG, triglyceride; TC, total cholesterol; HDL-C, high-density lipoprotein cholesterol; LDL-C, low-density lipoprotein cholesterol; WBC, white blood cell count; RBC, red blood cell count; Hb, hemoglobin; PLT, platelet count; NEU, neutrophils count; LYM, lymphocyte count; MONO, monocyte count; ALT, alanine transaminase; AST, aspartate aminotransferase; γ-GT, gamma-glutamyl transferase; ALP, alkaline phosphatase; ALB, albumin; GLB, globulin; T3, serum triiodothyronine; T4, thyroxine; TSH, thyroid-stimulating hormone; FT3, free triiodothyronine; FT4, free thyroxine.

All patients were further divided by sex and puberty. First, 581 male and 448 female patients were divided into the non-NAFLD group (male *vs.* female: n = 402, mean age, 10.53 ± 1.80 years, *vs.* n = 413, mean age, 8.93 ± 1.26 years) and the NAFLD group (male *vs.* female: n = 179, mean age, 11.18 ± 1.66 years, *vs.* n = 35, mean age, 10.27 ± 1.45 years; **Table 2**). Second, 573 patients in the prepuberty stage were divided into the non-NAFLD group (n = 504; male *vs.* female: n [%], 201 [39.88] *vs.* 303 [60.12]) and the NAFLD group (n = 69; male *vs.* female: n [%], 57 [82.61] *vs.* 12 [17.39]), while 456 patients in the early puberty stage were divided into the non-NAFLD group (n = 311; male *vs.* female: n [%], 201 [64.63] *vs.* 110 [35.37]) and the NAFLD group (n = 145; male *vs.* female: n [%], 122 [84.14] *vs.* 23 [15.86]) (**Table 3**). Third, 258 male and 315 female patients in the prepuberty stage were divided into the non-NAFLD (n = 201 and 303, respectively) and NAFLD (n = 57 and 12, respectively) groups. Also, 323 male and 133 female patients in the early puberty stage were divided into the non-NAFLD (n = 201 and 110, respectively) and NAFLD (n = 122 and 23, respectively) groups (**Tables 4**, **5**).

**Table 2:** Clinical characteristics in hospitalized children with obesity before the late puberty stage with or without NAFLD (divided by sex).

		Male			Female		
		Non-NAFLD (n = 402)	NAFLD (n = 179)	*p*-Value	Non-NAFLD (n = 413)	NAFLD (n = 35)	*p*-Value
Age (year)		10.53 ± 1.80	11.18 ± 1.66	0.000	8.93 ± 1.26	10.27 ± 1.45	0.000
BMI (kg/m^2^)		23.16 (21.57, 25.44)	27.47 (25.66, 29.90)	0.000	21.42 (20.12, 22.83)	28.04 (26.17, 29.85)	0.000
Blood pressure (n, %)	Normal	313 (77.86%)	103 (57.54%)	0.000	312 (75.55%)	19 (54.28%)	0.000
	Hypertension	70 (17.41%)	49 (27.38%)		81 (19.61%)	8 (22.86%)	
	Severe hypertension	19 (4.73%)	27 (15.08%)		20 (4.84%)	8 (22.86%)	
Birth weight (kg)		3.40 (3.00, 3.75)	3.40 (3.10, 3.60)	0.895	3.30 (3.00, 3.60)	3.25 (3.00, 3.75)	0.691
Delivery mode (n, %)	Natural	136 (33.83%)	73 (40.78%)	0.106	132 (31.96%)	13 (37.14%)	0.529
	Cesarean section	266 (66.17%)	106 (59.22%)		281 (68.04%)	22 (62.86%)	
Glucose and lipid metabolism							
HbA1c (%)		5.30 (5.20, 5.50)	5.40 (5.20, 5.60)	0.018	5.30 (5.10, 5.40)	5.50 (5.20, 5.60)	0.002
HOMA-IR		2.36 (1.38, 3.99)	3.56 (2.30, 5.20)	0.000	2.02 (1.19, 3.26)	4.62 (3.38, 6.53)	0.000
TG (mmol/L)		0.89 (0.69, 1.26)	1.23 (0.93, 1.65)	0.000	0.92 (0.68, 1.25)	1.29 (0.91, 1.73)	0.000
TC (mmol/L)		3.73 ± 0.65	4.06 ± 0.80	0.000	3.70 ± 0.66	3.78 ± 0.65	0.447
HDL-C (mmol/L)		1.22 (1.04, 1.41)	1.10 (0.94, 1.29)	0.000	1.24 (1.06, 1.43)	0.98 (0.88, 1.16)	0.000
LDL-C (mmol/L)		2.31 ± 0.59	2.62 ± 0.75	0.000	2.26 ± 0.63	2.36 ± 0.66	0.385
Blood routine							
WBC (×10^9^/L)		6.40 ± 1.54	6.92 ± 1.58	0.000	6.14 (5.23, 7.13)	6.01 (5.34, 7.48)	0.644
RBC (×10^12^/L)		4.65 (4.43, 4.87)	4.71 (4.51, 4.93)	0.019	4.55 ± 0.32	4.55 ± 0.32	0.907
Hb (g/L)		128.87 ± 9.80	130.79 ± 9.96	0.031	127.00 (122.00, 132.00)	130.00 (121.00, 135.00)	0.239
PLT (×10^9^/L)		291.92 ± 62.94	296.41 ± 68.71	0.441	288.57 ± 56.68	303.40 ± 53.22	0.136
NEU (×10^9^/L)		3.34 (2.67, 3.99)	3.65 (2.94, 4.27)	0.001	3.21 (2.60, 3.99)	3.21 (2.73, 4.38)	0.885
LYM (×10^9^/L)		2.33 (1.90, 2.65)	2.39 (2.01, 2.86)	0.015	2.36 ± 0.59	2.47 ± 0.68	0.673
MONO (×10^9^/L)		0.41 (0.33, 0.50)	0.45 (0.38, 0.53)	0.000	0.36 (0.30, 0.45)	0.38 (0.28, 0.51)	0.363
Liver function							
ALT (U/L)		15.00 (11.00, 20.00)	57.00 (26.00, 91.00)	0.000	12.00 (10.00, 15.00)	36.00 (18.00, 61.00)	0.000
AST (U/L)		20.00 (17.00, 24.00)	35.00 (23.00, 51.00)	0.000	19.00 (17.00, 22.00)	28.00 (18.00, 39.00)	0.000
γ-GT (U/L)		15.00 (13.00, 18.00)	29.00 (19.00, 47.00)	0.000	12.00 (10.00, 14.00)	23.00 (17.00, 31.00)	0.000
ALP (U/L)		238.00 (201.25, 289.00)	256.50 (218.00, 310.25)	0.004	273.17 ± 69.54	260.11 ± 77.70	0.291
ALB (g/L)		45.53 ± 3.01	46.09 ± 2.92	0.066	45.20 (43.70, 47.10)	46.10 (43.80, 47.10)	0.347
GLB (g/L)		24.92 ± 3.42	25.58 ± 3.32	0.031	24.12 ± 3.12	25.15 ± 4.62	0.201
Thyroid function							
T3 (nmol/L)		2.25 ± 0.48	2.22 ± 0.44	0.480	2.24 (1.97, 2.50)	2.17 (1.94, 2.49)	0.739
T4 (nmol/L)		107.15 ± 27.54	105.93 ± 24.49	0.614	103.90 ± 25.36	107.69 ± 20.69	0.390
TSH (μIU/ml)		2.41 (1.67, 3.43)	2.78 (1.90, 3.98)	0.013	2.25 (1.58, 3.19)	2.27 (1.71, 3.53)	0.705
FT3 (pmol/L)		6.40 (5.97, 6.92)	6.36 (5.76, 6.84)	0.064	6.49 (6.13, 6.96)	6.32 (5.85, 6.79)	0.098
FT4 (pmol/L)		15.80 (14.49, 17.40)	16.05 (14.25, 17.77)	0.731	15.94 (14.42, 17.31)	16.16 (14.61, 17.70)	0.294

NAFLD, non-alcoholic fatty liver disease; BMI, body mass index; HbA1c, glycosylated hemoglobin; HOMA-IR, homeostasis model assessment insulin resistance index; TG, triglyceride; TC, total cholesterol; HDL-C, high-density lipoprotein cholesterol; LDL-C, low-density lipoprotein cholesterol; WBC, white blood cell count; RBC, red blood cell count; Hb, hemoglobin; PLT, platelet count; NEU, neutrophils count; LYM, lymphocyte count; MONO, monocyte count; ALT, alanine transaminase; AST, aspartate aminotransferase; γ-GT, gamma-glutamyl transferase; ALP, alkaline phosphatase; ALB, albumin; GLB, globulin; T3, serum triiodothyronine; T4, thyroxine; TSH, thyroid-stimulating hormone; FT3, free triiodothyronine; FT4, free thyroxine.

**Table 3:** Clinical characteristics in hospitalized children with obesity before the late puberty stage with or without NAFLD (divided by puberty).

		Prepuberty			Early puberty		
		Non-NAFLD (n = 504)	NAFLD (n = 69)	*p*-Value	Non-NAFLD (n = 311)	NAFLD (n = 145)	*p*-Value
Sex (n, %)	Male	201 (39.88%)	57 (82.61%)	0.000	201 (64.63%)	122 (84.14%)	0.000
	Female	303 (60.12%)	12 (17.39%)		110 (35.37%)	23 (15.86%)	
BMI (kg/m^2^)		21.59 (20.15, 23.44)	26.75 (25.19, 29.28)	0.000	23.16 (21.71, 25.37)	27.95 (25.90, 30.39)	0.000
Blood pressure (n, %)	Normal	385 (76.39%)	40 (57.97%)	0.000	240 (77.17%)	82 (56.55%)	0.000
	Hypertension	94 (18.65%)	16 (23.19%)		57 (18.33%)	41 (28.28%)	
	Severe hypertension	25 (4.96%)	13 (18.84%)		14 (4.50%)	22 (15.17%)	
Birth weight (kg)		3.30 (3.00, 3.60)	3.35 (3.00, 3.50)	0.480	3.35 ± 0.49	3.42 ± 0.54	0.178
Delivery mode (n, %)	Natural	168 (33.33%)	29 (42.03%)	0.153	100 (32.15%)	57 (39.31%)	0.134
	Cesarean section	336 (66.67%)	40 (57.97%)		211 (67.85%)	88 (60.69%)	
Glucose and lipid metabolism							
HbA1c (%)		5.30 (5.10, 5.40)	5.40 (5.20, 5.60)	0.002	5.30 (5.10, 5.50)	5.40 (5.20, 5.60)	0.037
HOMA-IR		1.88 (1.14, 3.16)	3.24 (1.93, 4.48)	0.000	2.70 (1.63, 4.64)	4.13 (2.72, 5.61)	0.000
TG (mmol/L)		0.88 (0.67, 1.19)	1.20 (0.89, 1.51)	0.000	0.93 (0.71, 1.33)	1.31 (0.94, 1.69)	0.000
TC (mmol/L)		3.70 ± 0.64	4.01 ± 0.72	0.000	3.72 ± 0.69	4.02 ± 0.82	0.000
HDL-C (mmol/L)		1.23 (1.05, 1.41)	1.11 (0.97, 1.29)	0.001	1.22 (1.04, 1.43)	1.08 (0.90, 1.28)	0.000
LDL-C (mmol/L)		2.29 ± 0.59	2.57 ± 0.70	0.000	2.29 ± 0.63	2.59 ± 0.76	0.000
Blood routine							
WBC (×10^9^/L)		6.15 (5.37, 7.22)	6.71 (5.79, 7.75)	0.009	6.29 ± 1.43	6.82 ± 1.48	0.000
RBC (×10^12^/L)		4.55 (4.36, 4.77)	4.62 (4.47, 4.89)	0.011	4.66 ± 0.36	4.73 ± 0.37	0.066
Hb (g/L)		126.00 (121.00, 132.00)	130.00 (123.00, 134.50)	0.025	130.37 ± 9.89	131.24 ± 10.18	0.385
PLT (×10^9^/L)		292.48 ± 59.77	308.96 ± 75.43	0.085	286.59 ± 59.89	292.10 ± 61.07	0.365
NEU (×10^9^/L)		3.22 (2.63, 3.94)	3.40 (2.90, 4.32)	0.026	3.45 ± 1.08	3.66 ± 1.05	0.049
LYM (×10^9^/L)		2.39 (1.97, 2.71)	2.37 (1.94, 2.88)	0.497	2.25 (1.87, 2.56)	2.39 (2.02, 2.86)	0.001
MONO (×10^9^/L)		0.38 (0.31, 0.47)	0.45 (0.37, 0.55)	0.000	0.39 (0.32, 0.48)	0.45 (0.37, 0.52)	0.000
Liver function							
ALT (U/L)		13.00 (10.00, 17.00)	57.00 (26.50, 92.00)	0.000	13.00 (10.00, 18.50)	50.00 (24.50, 83.00)	0.000
AST (U/L)		20.00 (18.00, 24.00)	35.00 (25.50, 48.50)	0.000	18.00 (16.00, 22.00)	32.00 (22.00, 48.00)	0.000
γ-GT (U/L)		13.00 (11.00, 16.00)	24.00 (18.00, 46.00)	0.000	14.00 (11.00, 18.00)	29.00 (19.00, 43.00)	0.000
ALP (U/L)		254.00 (212.00, 311.00)	267.50 (234.75, 334.00)	0.085	257.67 ± 71.92	259.71 ± 77.69	0.783
ALB (g/L)		45.40 (43.90, 47.30)	46.00 (44.60, 48.15)	0.023	45.32 ± 2.81	45.88 ± 3.13	0.068
GLB (g/L)		24.20 ± 3.28	25.00 ± 3.51	0.063	25.02 ± 3.26	25.74 ± 3.56	0.034
Thyroid function							
T3 (nmol/L)		2.24 (1.98, 2.54)	2.21 (2.03, 2.58)	0.959	2.22 ± 0.41	2.20 ± 0.47	0.721
T4 (nmol/L)		107.48 ± 27.55	108.04 ± 23.06	0.870	102.29 ± 24.37	105.32 ± 24.28	0.227
TSH (μIU/ml)		2.41 (1.74, 3.51)	2.79 (1.84, 3.89)	0.205	2.18 (1.47, 3.18)	2.65 (1.87, 3.84)	0.000
FT3 (pmol/L)		6.49 (6.10, 6.98)	6.64 (5.85, 7.12)	0.907	6.39 (5.96, 6.89)	6.22 (5.68, 6.69)	0.008
FT4 (pmol/L)		16.04 ± 2.49	16.46 ± 2.51	0.234	15.50 (14.09, 16.83)	15.84 (14.17, 17.54)	0.287

NAFLD, non-alcoholic fatty liver disease; BMI, body mass index; HbA1c, glycosylated hemoglobin; HOMA-IR, homeostasis model assessment insulin resistance index; TG, triglyceride; TC, total cholesterol; HDL-C, high-density lipoprotein cholesterol; LDL-C, low-density lipoprotein cholesterol; WBC, white blood cell count; RBC, red blood cell count; Hb, hemoglobin; PLT, platelet count; NEU, neutrophils count; LYM, lymphocyte count; MONO, monocyte count; ALT, alanine transaminase; AST, aspartate aminotransferase; γ-GT, gamma-glutamyl transferase; ALP, alkaline phosphatase; ALB, albumin; GLB, globulin; T3, serum triiodothyronine; T4, thyroxine; TSH, thyroid-stimulating hormone; FT3, free triiodothyronine; FT4, free thyroxine.

**Table 4:** Clinical characteristics in hospitalized male children with obesity before the late puberty stage with or without NAFLD (subdivided by puberty).

		Male and prepuberty			Male and early puberty		
		Non-NAFLD (n = 201)	NAFLD (n = 57)	*p*-Value	Non-NAFLD (n = 201)	NAFLD (n = 122)	*p*-Value
BMI (kg/m^2^)		23.36 ± 3.17	27.43 ± 3.22	0.000	23.45 (22.01, 25.54)	27.85 (25.70, 30.45)	0.000
Blood pressure (n, %)	Normal	155 (77.11%)	34 (59.65%)	0.014	158 (78.61%)	69 (56.56%)	0.000
	Hypertension	34 (16.92%)	14 (24.56%)		36 (17.91%)	35 (28.69%)	
	Severe hypertension	12 (5.97%)	9 (15.79%)		7 (3.48%)	18 (14.75%)	
Birth weight (kg)		3.38 ± 0.52	3.27 ± 0.60	0.177	3.37 ± 0.49	3.41 ± 0.51	0.539
Delivery mode (n, %)	Natural	69 (34.33%)	24 (42.11%)	0.280	67 (33.33%)	49 (40.16%)	0.214
	Cesarean section	132 (65.57%)	33 (57.89%)		134 (66.67%)	73 (59.84%)	
Glucose and lipid metabolism							
HbA1c (%)		5.30 (5.15, 5.50)	5.4 (5.1, 5.6)	0.136	5.40 (5.18, 5.50)	5.40 (5.20, 5.60)	0.124
HOMA-IR		1.92 (1.15, 3.38)	2.91 (1.84, 4.40)	0.006	2.72 (1.71, 4.89)	3.90 (2.64, 5.31)	0.003
TG (mmol/L)		0.88 (0.68, 1.17)	1.15 (0.87, 1.46)	0.000	0.92 (0.70, 1.32)	1.28 (0.94, 1.74)	0.000
TC (mmol/L)		3.79 ± 0.66	4.02 ± 0.77	0.025	3.68 ± 0.65	4.08 ± 0.82	0.000
HDL-C (mmol/L)		1.26 ± 0.30	1.18 ± 0.30	0.066	1.22 (1.04, 1.41)	1.08 (0.91, 1.29)	0.000
LDL-C (mmol/L)		2.38 ± 0.58	2.60 ± 0.73	0.039	2.24 ± 0.59	2.63 ± 0.76	0.000
Blood routine							
WBC (×10^9^/L)		6.50 ± 1.62	6.96 ± 1.80	0.067	6.31 ± 1.45	6.91 ± 1.47	0.000
RBC (×10^12^/L)		4.54 (4.36, 4.79)	4.63 (4.47, 4.90)	0.036	4.75 ± 0.35	4.78 ± 0.36	0.509
Hb (g/L)		126.27 ± 8.80	128.81 ± 8.51	0.054	131.49 ± 10.07	131.73 ± 10.48	0.835
PLT (×10^9^/L)		296.12 ± 64.53	303.64 ± 77.49	0.460	287.72 ± 61.19	293.01 ± 64.23	0.462
NEU (×10^9^/L)		3.30 (2.64, 4.00)	3.41 (2.91, 4.26)	0.072	3.45 ± 1.05	3.73 ± 1.05	0.021
LYM (×10^9^/L)		2.41 ± 0.61	2.43 ± 0.58	0.816	2.27 (1.87, 2.55)	2.45 (2.01, 2.88)	0.002
MONO (×10^9^/L)		0.42 (0.33, 0.51)	0.46 (0.38, 0.57)	0.010	0.40 (0.32, 0.50)	0.45 (0.39, 0.53)	0.000
Liver function							
ALT (U/L)		15.00 (12.00, 20.00)	60.00 (27.50, 93.50)	0.000	14.00 (11.00, 20.00)	54.50 (25.75, 90.25)	0.000
AST (U/L)		21.00 (18.00, 25.00)	36.00 (25.50, 49.00)	0.000	19.00 (17.00, 22.00)	33.00 (22.00, 51.25)	0.000
γ-GT (U/L)		15.00 (13.00, 18.00)	24.50 (18.00, 47.50)	0.000	15.00 (12.25, 19.00)	31.00 (19.75, 47.00)	0.000
ALP (U/L)		229.00 (193.50, 270.50)	267.00 (233.50, 322.75)	0.000	262.00 ± 75.21	262.61 ± 77.02	0.943
ALB (g/L)		45.53 ± 3.12	46.15 ± 2.50	0.171	45.53 ± 2.90	46.06 ± 3.11	0.118
GLB (g/L)		24.65 ± 3.43	24.91 ± 3.04	0.605	25.19 ± 3.40	25.87 ± 3.40	0.079
Thyroid function							
T3 (nmol/L)		2.30 ± 0.52	2.24 ± 0.41	0.493	2.20 ± 0.42	2.21 ± 0.45	0.913
T4 (nmol/L)		113.03 ± 29.19	106.06 ± 24.47	0.101	101.15 ± 24.40	105.86 ± 24.61	0.103
TSH (μIU/ml)		2.49 (1.80, 3.87)	2.85 (1.89, 4.14)	0.467	2.33 (1.54, 3.17)	2.76 (1.90, 3.90)	0.002
FT3 (pmol/L)		6.43 (6.00, 6.93)	6.68 (5.83, 7.15)	0.747	6.37 (5.91, 6.92)	6.21 (5.68, 6.67)	0.038
FT4 (pmol/L)		16.37 ± 2.40	16.37 ± 2.66	0.998	15.42 (14.11, 16.75)	15.83 (13.97, 17.55)	0.263

NAFLD, non-alcoholic fatty liver disease; BMI, body mass index; HbA1c, glycosylated hemoglobin; HOMA-IR, homeostasis model assessment insulin resistance index; TG, triglyceride; TC, total cholesterol; HDL-C, high-density lipoprotein cholesterol; LDL-C, low-density lipoprotein cholesterol; WBC, white blood cell count; RBC, red blood cell count; Hb, hemoglobin; PLT, platelet count; NEU, neutrophils count; LYM, lymphocyte count; MONO, monocyte count; ALT, alanine transaminase; AST, aspartate aminotransferase; γ-GT, gamma-glutamyl transferase; ALP, alkaline phosphatase; ALB, albumin; GLB, globulin; T3, serum triiodothyronine; T4, thyroxine; TSH, thyroid-stimulating hormone; FT3, free triiodothyronine; FT4, free thyroxine.

**Table 5:** Clinical characteristics in hospitalized female children with obesity before the late puberty stage with or without NAFLD (subdivided by puberty).

		Female and prepuberty			Female and early puberty		
		Non-NAFLD (n = 303)	NAFLD (n = 12)	*p*-Value	Non-NAFLD (n = 110)	NAFLD (n = 23)	*p*-Value
BMI (kg/m^2^)		21.01 (19.86, 22.35)	27.53 (24.50, 30.32)	0.000	23.09 ± 2.66	28.27 ± 2.44	0.000
Blood pressure (n, %)	Normal	230 (75.91%)	6 (50.00%)	0.000	82 (74.55%)	13 (56.52%)	0.127
	Hypertension	60 (19.80%)	2 (16.67%)		21 (19.09%)	6 (26.09%)	
	Severe hypertension	13 (4.29%)	4 (33.33%)		7 (6.36%)	4 (17.39%)	
Birth weight (kg)		3.31 ± 0.50	3.10 ± 0.62	0.160	3.31 ± 0.48	3.47 ± 0.67	0.172
Delivery mode (n, %)	Natural	99 (32.67%)	5 (41.67%)	0.515	33 (30.00%)	8 (34.78%)	0.651
	Cesarean section	204 (67.33%)	7 (58.33%)		77 (70.00%)	15 (65.22%)	
Glucose and lipid metabolism							
HbA1c (%)		5.20 (5.10, 5.40)	5.50 (5.30, 5.60)	0.011	5.31 ± 0.36	5.42 ± 0.36	0.209
HOMA-IR		1.81 (1.13, 2.98)	3.81 (2.70, 4.79)	0.012	2.64 (1.53, 4.20)	4.79 (3.50, 9.30)	0.000
TG (mmol/L)		0.89 (0.66, 1.21)	1.27 (0.95, 1.83)	0.004	1.08 ± 0.48	1.40 ± 0.76	0.010
TC (mmol/L)		3.65 ± 0.62	3.98 ± 0.50	0.070	3.82 ± 0.75	3.68 ± 0.70	0.404
HDL-C (mmol/L)		1.24 (1.06, 1.41)	0.99 (0.91, 1.15)	0.011	1.27 ± 0.30	1.06 ± 0.28	0.002
LDL-C (mmol/L)		2.22 ± 0.60	2.39 ± 0.50	0.335	2.37 ± 0.70	2.34 ± 0.74	0.867
Blood routine							
WBC (×10^9^/L)		6.14 (5.29, 7.12)	6.42 (5.44, 8.92)	0.388	6.24 ± 1.39	6.34 ± 1.50	0.772
RBC (×10^12^/L)		4.57 ± 0.32	4.63 ± 0.29	0.507	4.51 ± 0.32	4.50 ± 0.34	0.896
Hb (g/L)		127.00 (122.00, 132.00)	130.5 (121.25, 138.75)	0.301	128.35 ± 9.24	128.70 ± 8.10	0.866
PLT (×10^9^/L)		290.05 ± 56.34	334.25 ± 61.36	0.008	284.55 ± 57.68	287.30 ± 41.29	0.827
NEU (×10^9^/L)		3.16 (2.57, 3.89)	3.21 (2.89, 4.56)	0.515	3.44 ± 1.14	3.29 ± 1.03	0.564
LYM (×10^9^/L)		2.40 ± 0.61	2.74 ± 0.91	0.230	2.25 ± 0.54	2.32 ± 0.47	0.568
MONO (×10^9^/L)		0.36 (0.30, 0.45)	0.39 (0.27, 0.49)	0.613	0.36 (0.31, 0.44)	0.37 (0.28, 0.51)	0.488
Liver function							
ALT (U/L)		12.00 (10.00, 14.00)	54.00 (23.25, 83.75)	0.000	11.00 (9.00, 15.00)	29.00 (14.00, 51.00)	0.000
AST (U/L)		20.00 (17.00, 23.00)	34.50 (23.75, 45.00)	0.000	17.00 (15.00, 21.00)	24.00 (18.00, 33.00)	0.000
γ-GT (U/L)		12.00 (10.00, 14.00)	23.50 (18.50, 31.50)	0.000	12.00 (10.00, 16.00)	22.00 (16.00, 31.00)	0.000
ALP (U/L)		281.60 ± 69.26	290.42 ± 63.00	0.664	249.75 ± 65.09	244.30 ± 81.14	0.727
ALB (g/L)		45.30 (43.80, 47.10)	46.90 (44.73, 49.65)	0.051	44.94 ± 2.61	44.90 ± 3.11	0.944
GLB (g/L)		23.90 ± 3.14	25.41 ± 5.33	0.351	24.72 ± 2.99	25.02 ± 4.33	0.685
Thyroid function							
T3 (nmol/L)		2.26 (1.98, 2.53)	2.42 (2.02, 2.92)	0.214	2.24 ± 0.39	2.16 ± 0.55	0.431
T4 (nmol/L)		103.75 ± 25.78	117.48 ± 11.02	0.001	104.31 ± 24.29	102.59 ± 22.83	0.754
TSH (μIU/ml)		2.30 (1.66, 3.20)	1.89 (1.72, 3.46)	0.662	2.13 (1.32, 3.19)	2.40 (1.68, 3.56)	0.226
FT3 (pmol/L)		6.54 (6.18, 7.02)	6.57 (5.86, 6.85)	0.701	6.42 ± 0.68	6.24 ± 0.69	0.273
FT4 (pmol/L)		15.82 ± 2.53	16.87 ± 1.67	0.195	15.76 (13.87, 17.17)	15.85 (14.59, 17.43)	0.703

NAFLD, non-alcoholic fatty liver disease; BMI, body mass index; HbA1c, glycosylated hemoglobin; HOMA-IR, homeostasis model assessment insulin resistance index; TG, triglyceride; TC, total cholesterol; HDL-C, high-density lipoprotein cholesterol; LDL-C, low-density lipoprotein cholesterol; WBC, white blood cell count; RBC, red blood cell count; Hb, hemoglobin; PLT, platelet count; NEU, neutrophils count; LYM, lymphocyte count; MONO, monocyte count; ALT, alanine transaminase; AST, aspartate aminotransferase; γ-GT, gamma-glutamyl transferase; ALP, alkaline phosphatase; ALB, albumin; GLB, globulin; T3, serum triiodothyronine; T4, thyroxine; TSH, thyroid-stimulating hormone; FT3, free triiodothyronine; FT4, free thyroxine.

### 3.2 Intergroup comparison of clinical characteristics

As shown in **Table 1**, the sex distribution was different between the two groups (*p* < 0.01). Male children with obesity revealed a significantly higher prevalence of NAFLD. Additionally, significant and progressive increases in age, BMI, HbA1c, HOMA-IR, TG, TC, LDL-C, WBC, RBC, Hb, NEU, LYM, MONO, ALT, AST, γ-GT, ALB, GLB, and TSH levels were found in the non-NAFLD and NAFLD groups (*p* < 0.05 and *p* < 0.01, respectively). Moreover, the incidence of hypertension was significantly higher whereas HDL-C and FT3 levels were significantly lower in children with obesity and NAFLD than in children with obesity and without NAFLD (*p* < 0.01).

As shown in **Table 2**, for both male and female children, similar to the total, a significant and progressive increase in age, BMI, the incidence of hypertension, HbA1c, HOMA-IR, TG, ALT, AST, and γ-GT and a significant decrease in HDL-C were found in the non-NAFLD and NAFLD groups (*p* < 0.05 and *p* < 0.01, respectively). Moreover, in male children, there was a significant and progressive increase in TC, LDL-C, WBC, RBC, Hb, NEU, LYM, MONO, GLB, and TSH in the non-NAFLD and NAFLD groups (*p* < 0.05 and *p* < 0.01, respectively). However, for both male and female children, the ALB and FT3 levels were not different between the two groups (*p* > 0.05), whereas the ALP level was significantly higher in male patients with obesity and NAFLD than in male children with obesity alone (*p* > 0.01).

As shown in **Table 3**, for both pre- and early puberty, consistent with the total, a significant and progressive increase in the prevalence of male children, BMI, the incidence of hypertension, HbA1c, HOMA-IR, TG, TC, LDL-C, WBC, NEU, MONO, ALT, AST, and γ-GT and a significant decrease in HDL-C were found in the non-NAFLD and NAFLD groups (*p* < 0.05 and *p* < 0.01, respectively). However, only in the prepuberty stage, as with the total, was a significant and progressive increase in RBC, Hb, and ALB found in the non-NAFLD and NAFLD groups (*p* < 0.05 and *p* < 0.01, respectively). Moreover, only in early puberty, as with total, were a significant and progressive increase in LYM, GLB, and TSH and a significant decrease in FT3 found in the non-NAFLD and NAFLD groups (*p* < 0.05 and *p* < 0.01, respectively).

As shown in **Tables 4**, **5**, even when subdivided by both sex and puberty, a significant and progressive increase in BMI, HOMA-IR, TG, ALT, AST, and γ-GT was still found in the non-NAFLD and NAFLD groups (*p* < 0.05 and *p* < 0.01, respectively). However, a significant increase in ALP was found in the non-NAFLD and NAFLD groups only in male children at the prepuberty stage (*p* < 0.01). Moreover, only in female children at the prepuberty stage was a significant elevation in platelet count and T4 levels found in the non-NAFLD and NAFLD groups (*p* < 0.01).

### 3.3 Identification of risk factors for NALFD in children with obesity

From total to stratification, all candidate variables were first included in the binary logistic regression analysis during the primary screening for potential risk factors. Next, multiple logistic regression was performed to determine independent risk factors.

**Figure 1:**
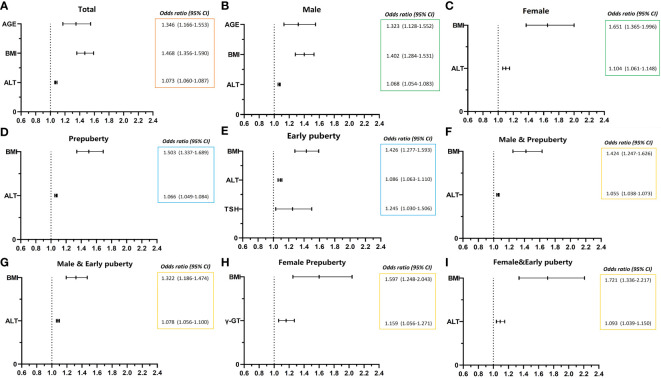
Risk factors of NAFLD of obese children before late puberty stage from logistic regression. NAFLD, nonalcoholic fatty liver disease. CI, confidence interval. BMI, body mass index. HOMA-IR, homeostasis model assessment insulin resistance index. HGB, hemoglobin. ALT, alanine transaminase. γ-GT, gamma glutamyl transferase. TSH, thyroid-stimulating hormone. **(A)** level of total; **(B, C)** stratified by gender; **(D, E)** stratified by puberty; **F–I**, stratified by both gender and puberty.

As presented in **Table 6** and **Figure 1**, age (OR, 1.346; 95% CI, 1.166–1.553; *p* < 0.001), BMI (OR, 1.468; 95% CI, 1.356–1.590; *p* < 0.001), and ALT level (OR, 1.073; 95% CI, 1.060–1.087; *p* < 0.001) were found to be three independent risk factors for NAFLD. However, HOMA-IR and Hb had no logistic regression relevance for pediatric NAFLD owing to their negative beta values.

**Table 6:** Risk factors of NAFLD in hospitalized children with obesity before the late puberty stage from logistic regression.

	Binary logistic regression				Multiple logistic regression			*p*-Value
	Beta	Standard error	Odds ratio (95% CI)	*p*-Value	Beta	Standard error	Odds ratio (95% CI)	
Total								
Age	0.403	0.104	1.496 (1.221–1.833)	0.000	0.297	0.073	1.346 (1.166–1.553)	0.000
BMI	0.378	0.052	1.459 (1.319–1.615)	0.000	0.384	0.041	1.468 (1.356–1.590)	0.000
HOMA-IR	−0.046	0.020	0.955 (0.919–0.993)	0.019	–0.044	0.019	0.957 (0.923–0.993)	0.020
Hb	−0.04	0.017	0.961 (0.93–0.993)	0.016	–0.035	0.013	0.965 (0.942–0.989)	0.005
ALT	0.074	0.019	1.076 (1.037–1.117)	0.000	0.071	0.007	1.073 (1.060–1.087)	0.000
Male								
Age	0.306	0.091	1.358 (1.135–1.624)	0.001	0.280	0.081	1.323 (1.128–1.552)	0.001
BMI	0.340	0.052	1.405 (1.269–1.556)	0.000	0.338	0.045	1.402 (1.284–1.531)	0.000
HOMA-IR	−0.068	0.034	0.935 (0.875–0.999)	0.045	–0.053	0.026	0.949 (0.902–0.997)	0.039
Hb	−0.071	0.020	0.931 (0.895–0.969)	0.000	–0.044	0.016	0.957 (0.928–0.987)	0.005
ALT	0.066	0.019	1.068 (1.029–1.108)	0.001	0.066	0.007	1.068 (1.054–1.083)	0.000
Female								
Age	0.492	0.233	1.636 (1.036–2.585)	0.035				
BMI	0.481	0.114	1.617 (1.292–2.023)	0.000	0.501	0.097	1.651 (1.365–1.996)	0.000
ALT	0.138	0.042	1.148 (1.056–1.247)	0.001	0.099	0.020	1.104 (1.061–1.148)	0.000
Prepuberty								
BMI	0.441	0.072	1.554 (1.348–1.791)	0.000	0.407	0.009	1.503 (1.337–1.689)	0.000
ALT	0.069	0.023	1.072 (1.025–1.121)	0.002	0.064	0.060	1.066 (1.049–1.084)	0.000
Early puberty								
BMI	0.339	0.069	1.404 (1.227–1.606)	0.000	0.355	0.056	1.426 (1.277-1.593)	0.000
ALT	0.113	0.031	1.119 (1.053–1.190)	0.000	0.083	0.011	1.086 (1.063-1.110)	0.000
TSH	0.331	0.116	1.392 (1.109–1.748)	0.004	0.219	0.097	1.245 (1.030-1.506)	0.023
FT3	−0.990	0.354	0.372 (0.186–0.743)	0.005				
Male and prepuberty								
BMI	0.412	0.081	1.51 (1.289–1.770)	0.000	0.354	0.068	1.424 (1.247–1.626)	0.000
ALT	0.052	0.024	1.054 (1.004–1.106)	0.032	0.054	0.009	1.055 (1.038–1.073)	0.000
Male and early puberty								
BMI	0.282	0.074	1.325 (1.147–1.531)	0.000	0.279	0.055	1.322 (1.186-1.474)	0.000
ALT	0.091	0.030	1.096 (1.033–1.162)	0.002	0.075	0.011	1.078 (1.056-1.100)	0.000
TSH	0.353	0.138	1.423 (1.085–1.866)	0.011				
Female and prepuberty								
BMI	0.633	0.228	1.883 (1.205–2.943)	0.005	0.468	0.126	1.597 (1.248–2.043)	0.000
γ-GT	−0.334	0.169	0.716 (0.514–0.997)	0.048	0.147	0.047	1.159 (1.056–1.271)	0.002
Female and Early puberty								
BMI	0.534	0.160	1.706 (1.246–2.337)	0.001	0.543	0.129	1.721 (1.336–2.217)	0.000
ALT	0.155	0.061	1.167 (1.036–1.315)	0.011	0.089	0.026	1.093 (1.039–1.150)	0.001

NAFLD, non-alcoholic fatty liver disease; CI, confidence interval; BMI, body mass index; HOMA-IR, homeostasis model assessment insulin resistance index; Hb, hemoglobin; ALT, alanine transaminase; γ-GT, gamma-glutamyl transferase; TSH, thyroid-stimulating hormone; FT3, free triiodothyronine.

As presented in **Table 6** and **Figure 1**, for male patients, age (OR, 1.323; 95% CI, 1.128–1.552; *p* < 0.01), BMI (OR, 1.402; 95% CI, 1.284–1.531; *p* < 0.001), and ALT level (OR, 1.068; 95% CI, 1.054–1.083; *p* < 0.001) were also found to be independent risk factors for NAFLD. However, in female patients, BMI (OR, 1.651; 95% CI, 1.365–1.996; *p* < 0.001) and ALT level (OR, 1.104; 95% CI, 1.061–1.148; *p* < 0.001) were found to be two independent risk factors for NAFLD, and age was no longer found to be an independent risk factor for NAFLD.

As presented in **Table 6** and **Figure 1**, BMI (OR, 1.503; 95% CI, 1.337–1.689; *p* < 0.001) and ALT (OR, 1.066; 95% CI, 1.049–1.084; *p* < 0.001) at the prepuberty stage were found to be two independent risk factors for NAFLD. Additionally, in early puberty, BMI (OR, 1.426; 95% CI, 1.277–1.593; *p* < 0.001) and ALT (OR, 1.086; 95% CI, 1.063–1.110; *p* < 0.001) were still found to be two independent risk factors for NAFLD. Additionally, during early puberty, the TSH level (OR, 1.245; 95% CI, 1.030–1.506; *p* < 0.05) was also an independent risk factor for NAFLD.

As presented in **Table 6** and **Figure 1**, after being graded by sex and puberty at the same time, BMI (male and prepuberty: OR, 1.424; 95% CI, 1.247–1.626; *p* < 0.001; male and early puberty: OR, 1.322; 95% CI, 1.186–1.474; *p* < 0.001; female and prepuberty: OR, 1.597; 95% CI, 1.248–2.043; *p* < 0.001; female and early puberty: OR, 1.721; 95% CI, 1.336–2.217; *p* < 0.01) remained an independent risk factor for NAFLD. Meanwhile, after being graded by both sex and puberty, ALT (male and prepuberty: OR, 1.055; 95% CI, 1.038–1.073; *p* < 0.001; male and early puberty: OR, 1.078; 95% CI, 1.056–1.100; *p* < 0.001; female and early puberty: OR, 1.093; 95% CI, 1.039–1.150; *p* < 0.001) remained an independent risk factor for NAFLD, except in prepuberty female children. Nevertheless, for prepuberty female children, γ-GT (OR, 1.159; 95% CI, 1.056–1.271; *p* < 0.01) was found to be a unique independent risk factor for NAFLD.

### 3.4 Evaluation of risk factors for NALFD in children with obesity

Independent risk factors were selected from the logistic regression analysis. A ROC curve analysis was performed to further evaluate the predictive ability of the independent risk factors for the early diagnosis of pediatric NAFLD.

**Figure 2:**
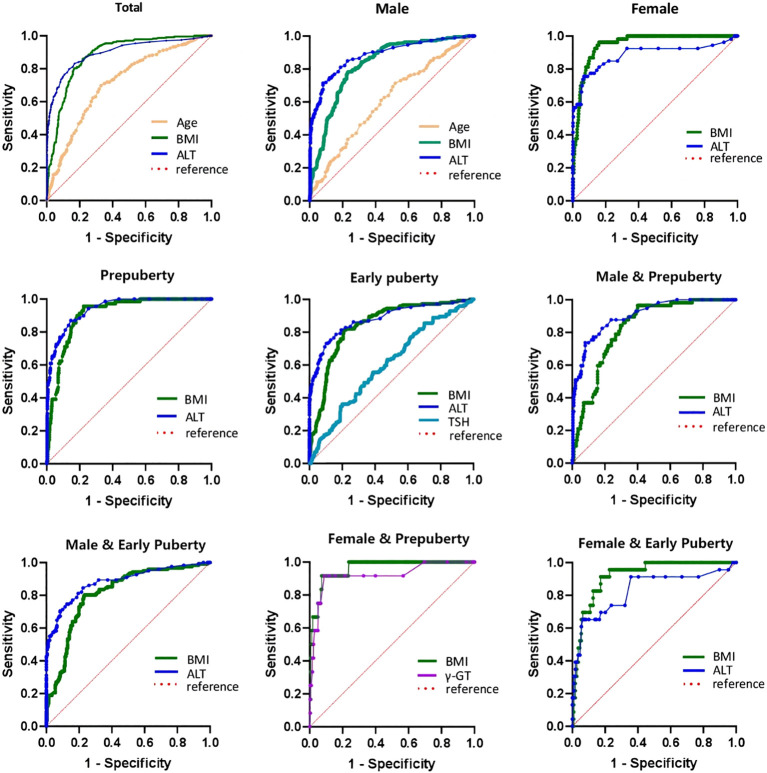
ROC curve of risk factors of NAFLD of obese children before late-puberty stage (Total and Stratification levels). ROC, receiver operating characteristics. NAFLD, nonalcoholic fatty liver disease. BMI, body mass index. HOMA-IR, homeostasis model assessment insulin resistance index. HGB, hemoglobin. ALT, alanine transaminase. γ-GT, gamma glutamyl transferase. TSH, thyroid-stimulating hormone.

As illustrated in **Table 7** and **Figure 2**, the ROC curve analysis of NAFLD in children with obesity before the late puberty stage revealed that BMI was a significant predictor. BMI presented optimal area under the ROC curve (AUROC) in three groups (female, 0.929–0.979; female and prepuberty, 0.921–0.998; female and early puberty, 0.865–0.970) and presented better AUROC in the other six groups (total, 0.861–0.907; male, 0.789–0.859; prepuberty, 0.880–0.973; early puberty, 0.802–0.880; male and prepuberty, 0.775–0.880; male and early puberty, 0.764–0.861).

**Table 7:** Receiver operating characteristic curve of risk factors to predict the occurrence of NAFLD in hospitalized children with obesity before the late puberty stage.

	AUC	Standard error	95% CI	Cutoff value	Sensitivity%	Specificity%	*p*-Value
Total							
Age	0.709	0.019	0.671–0.747	10.54	66.36	69.46	0.000
BMI	0.884	0.012	0.861–0.907	24.79	84.58	79.68	0.000
ALT	0.903	0.013	0.877–0.928	20.50	83.18	83.50	0.000
Male							
Age	0.603	0.025	0.553–0.652	10.67	68.16	51.00	0.000
BMI	0.824	0.018	0.789–0.859	25.56	77.65	77.00	0.000
ALT	0.886	0.016	0.855–0.917	30.50	71.51	91.75	0.000
Female							
BMI	0.954	0.013	0.929–0.979	24.23	94.29	87.14	0.000
ALT	0.885	0.038	0.811–0.959	26.00	68.57	94.90	0.000
Prepuberty							
BMI	0.908	0.015	0.880–0.973	23.76	95.65	77.53	0.000
ALT	0.940	0.012	0.916–0.965	20.50	86.96	85.29	0.000
Early puberty							
BMI	0.841	0.020	0.802–0.880	25.61	81.94	78.60	0.000
ALT	0.881	0.019	0.844–0.918	27.50	72.92	89.97	0.000
TSH	0.602	0.028	0.546–0.658	2.50	55.56	61.20	0.000
Male and prepuberty							
BMI	0.827	0.027	0.775–0.880	23.72	96.49	60.00	0.000
ALT	0.903	0.022	0.860–0.945	30.50	73.68	92.00	0.000
Male and early puberty							
BMI	0.812	0.025	0.764–0.861	25.56	80.33	76.50	0.000
ALT	0.883	0.021	0.843–0.923	27.50	74.59	87.50	0.000
Female and prepuberty							
BMI	0.959	0.020	0.921–0.998	24.23	91.67	92.74	0.000
γ-GT	0.919	0.049	0.822–1.000	17.50	91.67	91.09	0.000
Female and early puberty							
BMI	0.918	0.027	0.865–0.970	25.61	91.30	82.57	0.000
ALT	0.830	0.056	0.721–0.940	26.50	65.22	93.58	0.000

NAFLD, non-alcoholic fatty liver disease; AUC, area under the curve; CI, confidence interval; BMI, body mass index; ALT, alanine transaminase; γ-GT, gamma-glutamyl transferase; TSH, thyroid-stimulating hormone.

As illustrated in **Table 7** and **Figure 2**, excluding female children at the prepuberty stage, the ROC curve analysis demonstrated that ALT was another significant predictor. ALT presented optimal AUROC in six groups (total, 0.877–0.928; male, 0.855–0.917; prepuberty, 0.916–0.965; early puberty, 0.844–0.918; male and prepuberty, 0.860–0.945; male and early puberty, 0.843–0.923).

As illustrated in **Table 7** and **Figure 2**, three other significant predictors were distinctive to certain groups: age for the total group and male group, TSH for the early puberty group, and γ-GT for the female and prepuberty groups. Age showed good AUROC in the total group, 0.709 (0.671–0.747); in contrast, γ-GT showed remarkable AUROC in the female and prepuberty groups, 0.919 (0.822–1.000).

## 4 Discussion

In this study, we targeted children with obesity because of the global NAFLD epidemic (1). Although teenagers have a higher risk of NAFLD than young children, the children suffering the most from NAFLD are aged 10–13 years (17, 18). Italian case reports suggest a lower age of onset (10–11 years) (19–21). Therefore, in the current study, we chose those aged before the late puberty stage from among the target participants and conducted a retrospective case analysis to evaluate the adaptability of some known risk factors for NAFLD in children in central China.

The present study reported that pediatric NAFLD is more likely to occur in older children and male children; pediatric NAFLD has clinical characteristics of elevated BMI and increased incidence of hypertension; pediatric NAFLD has alterations in biochemical characteristics of glucose and lipid metabolisms (low HDL-C, high TG, TC, LDL-C, and HbA1c), routine blood investigations (high WBC, NEU, LYM, MONO, RBC, and Hb), liver function tests (high ALT, AST, γ-GT, ALB, and GLB), and thyroid function tests (low FT3 and high TSH). The majority of the above significant factors have been reported and accepted (6, 22–28). However, the correlation between the prevalence of pediatric NAFLD and blood cell levels is unclear because there is little research on this topic. In this study, after logistic regression analysis, age remained significant for male children rather than female children. The specific cutoff is 10.67 years, supporting a peak onset age of 10–11 years (19–21).

Furthermore, stratified analysis was performed to verify the aforementioned significant variables at the total level. First, BMI, HOMA-IR, TG, ALT, AST, and γ-GT levels were always statistically significant regardless of stratification. Second, the female children tested negative for alterations in TC, LDL-C, and all thyroid function tests. Third, all thyroid function tests were negative for alterations in the patients at the prepuberty stage. Fourth, HbA1c levels were negative when subdivided according to puberty in male children. Fifth, the incidence of hypertension and HbA1c were negative in female children in the early puberty stage.

It has been demonstrated that female children are more sensitive to TG than cholesterol. This finding is in accordance with those of 29–32, who reported that one of the components related to the association between NAFLD and metabolic syndrome risk factors in female children was hypertriglyceridemia. Our results also showed that biomarkers reflecting hypothyroidism may not be sensitive to female sex and age at the prepuberty stage. Thyroid hormones influence all major metabolic pathways. Thyroid dysfunction, especially hypothyroidism, has been associated with IR, dyslipidemia, and obesity, all of which play important roles in the development of NAFLD (29). Pacifico et al. and Kaltenbach et al. reported that increased TSH levels could be an important risk factor for the development and progression of NAFLD in children and adolescents with obesity; however, in contrast to those of our study, these results did not change after adjusting for sex and stage of puberty (29, 33). In this study, after logistic regression analysis, TSH only remained significant at the early puberty stage, and the specific cutoff was 2.5 U/ml, which is in the range of 2.31–3.22 U/ml, as suggested in the study of Kaltenbach et al. (33).

The results of the logistic regression analysis indicated that BMI played a dominant role in all risk factors because it maintained significance for both the total and all stratified levels. Overall, the risk of NAFLD in children with obesity increased by 46.8% for every unit increase in BMI. After stratification, the OR of BMI of the four groups (female, prepuberty, female and prepuberty, and female and early puberty) exceeded the total level, and the maximal OR was found in the female and early puberty group, 1.721 (95% CI, 1.336–2.217), which means that for obese female children before late puberty, BMI might be more crucial for risk exposure to NAFLD. The sex differences in the prevalence of NAFLD may be due to the fat distribution and the protective effects of estrogen (21). In a study by Cohen et al. (34), BMI was significantly higher in female children than in male children; Alkassabany YM et al. also reported no significant correlation between gender and NAFLD (35). However, in our study, the BMI cutoff value for female children was lower than for male children (24.23 *vs.* 25.56).

This study also revealed that ALT is important because of its high frequency of emergence. Overall, the risk of NAFLD in children with obesity increased by 7.3% for every unit increase in ALT. After stratification, four groups (female, early puberty, male and early puberty, and female and early puberty) showed a higher OR, and the female group showed the maximal OR of 1.104 (95% CI, 1.061–1.148), which means that in the early puberty age stage, ALT might be a necessary risk factor for NAFLD, especially in female children. This finding is in accordance with those of many previous studies (27, 31, 35–39). Villanueva-Ortega et al. suggested that although ALT is the only predictor of NAFLD in male children, it does not have the same diagnostic efficiency as it does in female patients (27). Wiegand et al. found that the association of significantly elevated ALT with age supports the hypothesis that an increase in sex hormones at the prepuberty stage may be important in the predisposition to pediatric NAFLD (35). In addition, the results of the ROC analysis showed that ALT was a better predictor of pediatric NAFLD than BMI. The North American guidelines use an ALT of >52 U/L for male children and an ALT of >44 U/L for female children, and the European guidelines use an elevated ALT of >45 U/L for both sexes (40, 41), but different from them, the cutoff value in our study was 30.5 U/L and 26 U/L for male and female children, respectively.

In summary, we conclude that BMI, ALT, and age are risk factors for NAFLD in children with obesity before the late puberty stage. BMI had the greatest exposure risk for NAFLD, and ALT had the highest predictive value for the diagnosis of NAFLD. At the stratified level, for exposure risk, age was specific to the male sex, TSH was specific to the early puberty stage, and γ-GT was specific to the female sex plus the prepuberty stage. On a stratified level, for the female sex, even with age stratification, BMI rather than ALT has a better ability to diagnose NAFLD.

In the end, we are aware of the limitation of the present study. First, some well-known risk factors for pediatric NAFLD were missing, such as lifestyle (dietary factors and physical inactivity), ethnicity, genetic factors, and maternal factors (metabolic disease, metabolic level during pregnancy, breastfeeding, etc.) (42, 43). Second, there is a popular study about the new biomarkers to monitor NAFLD, such as Sirtuin 1, CK-18, and more genetic and epigenetic factors (42, 44, 45). Due to retrospective nature of the studies, no data were available. Third, more accurate diagnostic methods for pediatric NAFLD, such as nuclear magnetic resonance and liver biopsy, were not selected. Fourth, the design of the control group could be more comprehensive, including healthy children, overweight and non-obese patients, patients in late puberty, and non-alcoholic fatty hepatitis (NASH) patients. Fifth, since the global coronavirus disease-2019 pandemic occurred during the data collection period, it is not clear whether this event had an impact on the disease.

### 4.1 Future perspective

The debate existed for many years that Calls to rename NAFLD to Metabolic [Dysfunction-] Associated Liver Disease (MAFLD) (46–48) has existed for many years. The Chinese Society of Hepatology (CSH) endorsed the proposal of changing the term NAFLD to MAFLD in 2021 (49). Clinical diagnosis of MAFLD would be based upon hepatic steatosis (liver biopsy, imaging, or blood biomarker evidence) and at least one of three criteria (obesity/overweight, type 2 diabetes (T2D), or evidence of at least two metabolic abnormalities). We have reviewed our data, the diagnosis is a basic one. However, this a retrospective study, and the design of this article predates the renaming statement for nonalcoholic fatty liver disease. We can consider that using the new nomenclature in future studies for MAFLD would benefit the patient’s understanding and management of the disease and be better for identifying significant hepatic diseases. Moreover, so far, the discussion primarily addressed adult patients and now awaits pediatric contribution (47).

To this date, there is no pharmacological drug registered for the treatment of pediatric NAFLD (49). Proposed pharmacological therapies include insulin sensitizers, probiotics, anti-inflammatory agents, lipid-lowering drugs, and vitamins. Recently, using natural products as an alternative approach in the treatment of NAFLD has drawn increasing attention. Among the most studied substances in the literature, the following molecules were chosen: spirulina, oleuropein, garlic, berberine, resveratrol, curcumin, ginseng, glycyrrhizin, coffee, cocoa powder, epigallocatechin-3-gallate, and bromelain (48, 50, 51). However, there are many examples of hepatotoxicity induced by herbal remedies (42, 52, 53), and the causes are different, such as impurities, batch-to-batch variability, misidentification and/or labeling, and different source of used production materials. That said, some herbal products should be consumed with caution due to their hepatotoxicity.

It is noteworthy that the excess visceral adipose tissue (VAT) may be more important than BMI (54). Obese persons with excess VAT, or abdominal obesity, are at higher risk for metabolic syndrome components than those whose fat is located predominantly in the lower body subcutaneously. However, our research lacks data on waist circumference and visceral fat, so it cannot be compared with BMI, which will be an important point in our next research.

## Data availability statement

The original contributions presented in the study are included in the article/supplementary material. Further inquiries can be directed to the corresponding authors.

## Ethics statement

The studies involving humans were approved by the Medical Ethics Committee of the Wuhan Children’s Hospital. The studies were conducted in accordance with the local legislation and institutional requirements. The human samples used in this study were acquired from another research group. Written informed consent for participation was not required from the participants or the participants’ legal guardians/next of kin in accordance with the national legislation and institutional requirements.

## Author contributions

LishZ and XC conceived and designed the study. LinlZ, PC, LingZ, WY, XY, YW, XX, and SY collected the clinical data. LishZ, LinlZ, and HM performed the data statistics. LishZ, LinlZ, WY, HX, and HQ wrote the paper. LishZ, LingZ, and HD reviewed and edited the manuscript. All authors read and approved the manuscript.
